# Geographic Variation in Heart Failure Mortality and Its Association With Hypertension, Diabetes, and Behavioral-Related Risk Factors in 1,723 Counties of the United States

**DOI:** 10.3389/fpubh.2018.00132

**Published:** 2018-05-07

**Authors:** Longjian Liu, Xiaoyan Yin, Ming Chen, Hong Jia, Howard J. Eisen, Albert Hofman

**Affiliations:** ^1^Department of Epidemiology and Biostatistics, Drexel University Dornsife School of Public Health, Philadelphia, PA, United States; ^2^Department of Epidemiology, Harvard T.H. Chan School of Public Health, Boston, MA, United States; ^3^Division of Endocrinology, Diabetes and Metabolism, Perelman School of Medicine at University of Pennsylvania, Philadelphia, PA, United States; ^4^Department of Cardiology, First Affiliated Hospital of Chongqing Medical University, Chongqing, China; ^5^Department of Epidemiology and Biostatistics, Southwest Medical University School of Public Health, Luzhou, Sichuan, China; ^6^Division of Cardiology, Department of Medicine, Drexel University College of Medicine, Philadelphia, PA, United States

**Keywords:** heart failure, mapping, risk factors, mortality, United States

## Abstract

**Background and objectives:**

Studies that examined geographic variation in heart failure (HF) and its association with risk factors at county and state levels were limited. This study aimed to test a hypothesis that HF mortality is disproportionately distributed across the United States, and this variation is significantly associated with the county- and state-level prevalence of high blood pressure (HBP), diabetes, obesity and physical inactivity.

**Methods:**

Data from 1,723 counties in 51 states (including District of Columbia as a state) on the age-adjusted prevalence of obesity, physical inactivity, HBP and diabetes in 2010, and age-adjusted HF mortality in 2013–2015 are examined. Geographic variations in risk factors and HF mortality are analyzed using spatial autocorrelation analysis and mapped using Geographic Information System techniques. The associations between county-level HF mortality and risk factors (level 1) are examined using multilevel hierarchical regression models, taking into consideration of their variations accounted for by states (level 2).

**Results:**

There are significant variations in HF mortality, ranging from the lowest 11.7 (the state of Vermont) to highest 85.0 (Mississippi) per 100,000 population among the 51 states. Age-adjusted prevalence of obesity, physical inactivity, HBP, and diabetes are positively and significantly associated with HF mortality. Multilevel analysis indicates that county-level HF mortality rates remain significantly associated with diabetes (β = 2.7, 95% CI: 1.7–3.7, *p* < 0.0001), HBP (β = 3.6, 2.1–5.0, *p* < 0.0001), obesity (β = 0.9, 0.6–1.3, *p* < 0.0001), and physical inactivity (β = 1.2, 0.8–1.5, *p* < 0.0001) after controlling for gender, race/ethnicity, and poverty index. After further controlling obesity and physical inactivity in diabetes and HBP models, the effects of diabetes (β = 1.0, −0.3 to 2.3, *p* = 0.12) and HBP (β = 2.4, 0.9–3.9, *p* = 0.003) on HF mortality had a considerable reduction.

**Conclusion:**

HF mortality disproportionately affects the counties and states across the nation. The geographic variations in HF morality are significantly explained by the variations in the prevalence of obesity, physical inactivity, diabetes, and HBP.

## Introduction

Heart failure (HF), one of the major forms of cardiovascular diseases, is a complex clinical syndrome that results in the impairment of heart’s ability to fill or to pump out blood (1, 2). HF has posed a serious public health problem, with a prevalence of over 5.8 million in the United States (U.S.) and over 23 million worldwide. HF is rising nationally and internationally (1, 3). About half of people who develop HF die within 5 years of diagnosis. HF costs the U.S. an estimated $30.7 billion each year, which includes the cost of health care services, medications to treat HF, and missed days of work (1, 3–7). The risk factors for HF include diseases that damage the heart, such as coronary heart disease, high blood pressure (HBP), diabetes and unhealthy behaviors (i.e., unhealthy dietary patterns, physical inactivity, smoking tobacco, etc.) (7). Most studies of the associations between risk factors and HF have been conducted at a personal level, which adds essential information to modify individual health behaviors and improve clinical treatments (5, 8–11). However, control of risk factors and diseases at population and community levels across the counties and states would play a critical role in moving toward the goal of healthy counties and states. Furthermore, health policy and population-based prevention programs have been designed and made at county- and state levels. In the last two decades, HF has become a new epidemic in the nation and worldwide (3, 6, 12). However, studies that examined geographic variations in HF and its determinants across the counties and states were insufficient (6). In this study, we aimed to test a hypothesis that a significant geographic disparity in HF mortality exists across the nation, and this geographic disparity is significantly associated with four preventable behavior- and disease-related risk factors. To test this hypothesis, we used data from three nationally representative data sources to examine the geographic variations in HF mortality and to examine the associations between the risk of HF mortality and the prevalence of obesity, physical inactivity, diabetes and HBP at the county- and state levels. Findings from the study may add new evidence to the body of the literature and provide substantial evidence to health policymakers and practitioners for control of HF at community and population levels.

## Study Design and Methods

### Study Design

**Figure 1:**
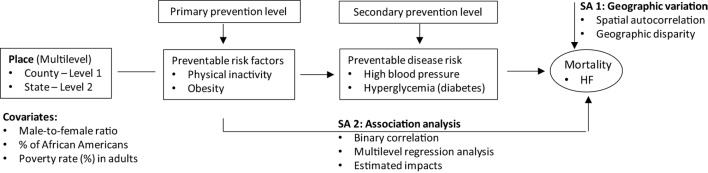
Conceptual model and analysis framework for specific aims (SA) 1 and 2.

To address the geographic variations in risk factors and HF mortality, we applied spatial and ecological analyses approaches. The current knowledge suggests that one of the biological pathways by which behavior risk factors increase the risk of heart diseases may go through an increased risk for diabetes and HBP (2, 6, 13–15). On the basis of a temporal causal-effect association between exposures and outcomes, we examined the association for risk factors that were measured in 2010 (diabetes, HBP, obesity and physical inactivity), and outcomes (i.e., HF mortality) that were measured in 2013–2015. Figure 1 illustrates the study conceptual model and data analysis framework.

### Study Population and Data

In the study, we collected data from 51 states (including the District of Columbia as a state) in the U.S. County-level sex-specific and age-adjusted HF mortality rates were collected from the U.S. CDC WONDER (Wide-ranging Online Data for Epidemiologic Research) (16). Data for risk factors were collected from U.S. CDC County Data report, the National Health and Nutrition Examination Surveys (NHANES), and the national Behavior Risk Factor Surveillance System (BRFSS) (Table S1 in Supplementary Material) (17–19). All data used in the study are de-identified and released publicly by the U.S. CDC for researchers (16–19).

#### Outcomes

County- and state-level age-adjusted HF mortality rates were estimated in residents aged 35 and older for the period 2013–2015 using direct standardization method. The U.S. 2000 standard population was used in the direct standardization (16). We included those who were aged 35 and older because HF mainly affects adults and older people. We calculated an average of 3-year HF mortality to have a relatively stable and representative estimate of HF mortality for each county because as compared with the other major forms of cardiovascular diseases (coronary heart disease and stroke), HF mortality rate was much lower. For example, the average age-adjusted mortality rates were 209.8 per 100,000 population for coronary heart disease, 80.4 per 100,000 population for stroke, and 51.6 per 100,000 population for HF during the period of 2013–2015 (16).

#### Exposures

We focus on two groups of exposures: (1) Behavior-related factors, including county- and state-level age-adjusted prevalence of obesity and physical inactivity. Obesity is defined as body mass index [weight (kg)/height (m)*height (m) ≥ 30 kg/m^2^]. Physical inactivity is defined as a “no” response to the question, “During the past month, other than your regular job, did you participate in any physical activities or exercises such as running, calisthenics, golf, gardening, or walking for exercise?” (19) (2) Health conditions for clinical treatments, including the age-adjusted prevalence of HBP and diabetes. We focused on these four risk factors because they have high frequency and are largely preventable at community and population levels. Data for the county- and state-level age-adjusted prevalence of diabetes, obesity and physical inactivity were collected from the U.S. CDC County Data report (17). However, because data for the county- and state-level age-adjusted prevalence of HBP was not directly available from the CDC County Data report, we applied a multiple stage regression prediction method to estimate the prevalence of HBP using data from BRFSS and NHANES (18, 19). This estimation method is briefly described below. The details have been discussed in Ezzati and colleagues’ report (20).

##### Estimate of the State-Level Prevalence of HBP

We first estimated the state-level prevalence of HBP as BRFSS has the most data for the measures of HBP at the state level, but it has no exact measures of systolic and diastolic blood pressure (SBP and DBP). If we estimated the prevalence of HBP using the data from BRFSS only, we would miss cases for those who did not know whether they had HBP due to without exact measures of SBP and DBP. To estimate SBP and DBP, we applied multi-stage regression analysis approach (20). We first estimated the associations (assessed by regression coefficients, β*_i_*s) between BP (SBP and DBP) and predictors (i.e., BP = β_1_*age + β_2_*BMI + β_3_*DM + β_4_*Physical inactivity) using these measured variables in NHANES (SBP, DBP, age, BMI, diabetes, and physical inactivity). We then applied the estimated β_1–4_ by using data from NHANES to estimate SBP and DBP using data from BFRSS.

##### Estimate of the County-Level Prevalence of HBP

Behavior Risk Factor Surveillance System has personal health behavior and health condition data by states, but not by county. Therefore, we further estimated the county-level prevalence of HBP using the estimated state-level prevalence of HBP and using data on the prevalence of obesity, physical inactivity, and diabetes from the CDC County Data system. In the first step, we calculated the associations (assessed by regression coefficients, β*_i_*) of state-level HBP (estimated from the above section) with state-level obesity, diabetes and physical inactivity using data from BRFSS (i.e., HBP_BR-NHANES_S_ = *a* + β_BR_SO_*X_BR_SO_ + β_BR_SDM_*X_BR_SDM_ + β_BR_SPIA_*X_BR_SPIA_), where *X*_BR_ represents data from BRFSS. HBP_BR-NHANES_S_ represents the estimated HBP from both BRFSS and NHANES (see the above section), *X*_BR_SO_, *X*_BR_SDM_, and *X*_BR_SPIA_ represent the state-level prevalence of obesity, diabetes, and physical inactivity from BRFSS. The estimated regression coefficients of obesity (β_BR_SO_), diabetes (β_BR_SDM_), and physical inactivity (β_BR_SPIA_) were then applied to estimate county-level HBP using country-level obesity (*X*_CDC_CO_), diabetes (*X*_CDC_CDM_), and physical inactivity (*X*_CDC_CPIA_) represent data from CDC County Data system (i.e., HBP_ES_C_ = *a* + β_BR_SO_*X_CDC_CO_ + β_BR_SDM_*X_CDC_CDM_ + β_BR_SPIA_*X_CDC_CPIA_), where HBP_ES_C_ represents the estimated county-level prevalence of HBP.

#### Covariates

In the multilevel hierarchical linear regression models, we tested the associations between risk factors and HF mortality, adjusting demographic factors (i.e., male-to-female ratio and % of African Americans), and socioeconomic status (assessed by poverty rate in adults aged 18 and older), because these factors are the strongest confounders in the study of the associations between the predictors and outcomes of the study (21).

### Statistical Analysis

A serial analysis was conducted. In the first group analysis, we described the characteristics of the study samples by regions (Northeast, Midwest, South, and West) and tested the difference in the characteristics using analysis of variance for continuous variables, and chi-square test for categorical variables. Geographic variations in the study variables of interest were examined using spatial autocorrelation and mapped using the Geographic Information System technique (GIS, ArcGIS version 12) (22). The spatial autocorrelation is a measure of the degree to which a set of spatial features (i.e., latitude and longitude) and their associated data values (i.e., risk factors and HF mortality) tend to be clustered together (positive spatial autocorrelation) or dispersed geographically (negative spatial autocorrelation). Two statistics, Moran’s *I*, and Geary’s *C* (i.e., spatial correlation coefficients) are used to evaluate the spatial correlation. Moran’s *I* ranges from −1 to +1, where values between 0 and +1 indicate a positive association between variables, and values between 0 and −1 indicate a negative association and 0 indicates there is no correlation between variables. Geary’s *C* is always positive and usually ranges from 0 to (+2), where a positive autocorrelation is less than 1, and a negative autocorrelation is greater than 1. SAS procedure of variogram was used to test spatial autocorrelation (23–25).

In the second group analysis, we examined associations of age-adjusted HF mortality with age-adjusted prevalence of hypertension, diabetes, physical inactivity, and obesity using correlation analysis (assessed by correlation coefficients and its 95% confidence intervals) (26).

In the third group analysis, we estimated the impacts of county-level risk factors (obesity, physical inactivity, HBP, and diabetes) on the age-adjusted HF mortality (level 1) with adjusting covariates (male-to-female ratio, % of African Americans and poverty rate) and considering the variations accounted for by states (level 2) using multilevel hierarchical linear regression analysis techniques (27–29). We evaluated the fitness of models using Akaike information criterion (AIC), the smaller value of the AIC, the better a model is fitted. We calculated Pseudo *R*^2^ to test the proportional reduction in residual variance between two nested models when adding additional predictors and covariates (30, 31).

Finally, we repeated the above correlation analyses by transferring the study variables of interest to *z*-scores to use data with standardized normal distributions (32). The results are similar to those using non-transformed variables. Therefore, to easily interpret the findings, we reported the results of the analysis using the non-transformed datasets.

All data analyses were analyzed and are presented separately for men and women because sex is considered as a biological variable. However, the mapping presentations are presented for sex combined because they had similar distributions in the study samples. In multilevel models, we initially analyzed by sex, and then for a combined sample of both sexes while adjusting for sex (male-to-female ratios) to present a summary finding. In the study, we were unable to calculate county-level age-sex-adjusted mortality rates, because we had no individual-level data by age and sex. We used ArcGIS (version 10.31, Esri, Redlands, CA, USA) and SAS (version 9.4, SAS Institute, Cary, NC, USA) in mapping and statistical analyses. A two-sided *p*-value ≤ 0.05 was considered as having statistical significance.

## Results

### Characteristics of the Study Samples by Regions

Table 1 shows that of the four U.S. Census regions, residents who lived in the counties located in the South had the highest age-adjusted HF mortality (59.6 per 100,000 population), followed by those who lived in the Midwest (50.3 per 100,000), the Northeast (36.3 per 100,000), and West (35.4 per 100,000) in the period 2013–2015. A similar distribution of risk factors was seen. Residents who lived in the counties located in the South had the highest age-adjusted prevalence of diabetes (10.8%), HBP (32.9%), obesity (32.0%), and physical inactivity (29.1%). Meanwhile, the South had the highest male-to-female ratio (99.4%), the highest proportion of African Americans (17.4%), the highest poverty rate in adults aged 18 and older (27.2%) than the other three regions.

**Table 1:** Characteristics of study population by regions.

County-level variables of interest	All total sample (*N* = 1,723) Mean (min–max)	By regions							
		Northeast (*N* = 194) Mean (SD)	Midwest (*N* = 486) Mean (SD)	West (*N* = 193) Mean (SD)	South (*N* = 850) Mean (SD)	*p*-Values for regional and overall diff			
						Regional diff (ref: South)			Overall
						NE vs. S	MW vs. S	W vs. S	
**HF mortality (per 100,000 population)**									
HF mortality in both gender	51.6 (5.2–367.0)	36.3 (12.2)	50.3 (24.5)	35.4 (19.5)	59.6 (32.2)	<0.0001	<0.0001	<0.0001	<0.0001
HF mortality in men	58.9 (6.0–261.4)	42.9 (13.7)	58.2 (25.7)	36.7 (17.8)	70.5 (42.1)	<0.0001	<0.0001	<0.0001	<0.0001
HF mortality in women	48.1 (3.7–304.8)	34.1 (12.5)	49.0 (24.9)	31.3 (18.8)	56.1 (32.3)	<0.0001	0.001	<0.0001	<0.0001

**Prevalence of factors in both gender (%)**									
Diabetes	9.6 (3.9–15.9)	8.4 (1.2)	8.8 (1.3)	7.6 (1.4)	10.8 (1.6)	<0.0001	<0.0001	<0.0001	<0.0001
High blood pressure	31.5 (25.1–38.7)	29.8 (1.4)	30.7 (1.5)	29.0 (1.5)	32.9 (1.9)	<0.0001	<0.0001	<0.0001	<0.0001
Obesity rate	30.4 (13.9–47.6)	27.2 (3.8)	30.6 (3.0)	25.6 (4.3)	32.0 (4.0)	<0.0001	<0.0001	<0.0001	<0.0001
Physical inactivity rate	26.5 (10.4–43.1)	23.9 (3.4)	25.7 (4.0)	19.4 (3.7)	29.1 (4.6)	<0.0001	<0.0001	<0.0001	<0.0001

**Demographics and economic status**									
Male-to-female ratio, %	97.1 (93.4–108.5)	94.7 (1.0)	96.9 (1.3)	97.2 (3.5)	99.4 (1.6)	<0.0001	0.306	<0.0001	<0.0001
% of African Americans	10.4 (0.2–82.3)	5.3 (6.5)	3.8 (5.2)	2.0 (2.3)	17.4 (16.7)	<0.0001	<0.0001	<0.0001	<0.0001
Poverty rate in adults aged ≥18, %	23.8 (3.8–57.1)	17.9 (6.4)	20.7 (6.9)	22.1 (7.5)	27.2 (8.5)	<0.0001	<0.0001	<0.0001	<0.0001

*HF, heart failure*.

*Annual average age-adjusted mortality (100,000 population) in residents aged ≥35 in the periods of 2013–2015*.

*Age-adjusted prevalence of diabetes, high blood pressure, obesity, and physical inactivity were estimated in adults aged ≥18*.

*Demographic and economic status (assessed by the % of poverty rate) variables were estimated in 2010 census*.

### Mapping the Variations in Risk Factors and HF Mortality Across the States and Counties

**Figure 2:**
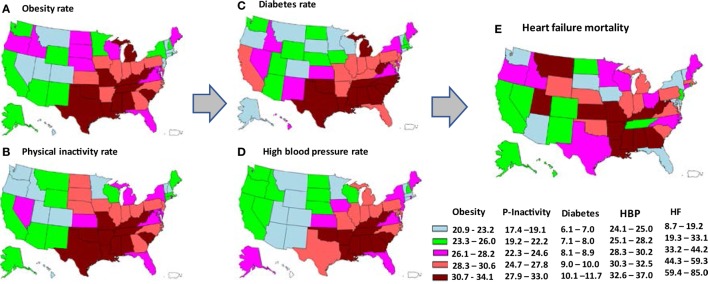
State-level variations in age-adjusted prevalence of obesity **(A)**, physical inactivity **(B)**, diabetes **(C)**, high blood pressure (HBP) **(D)**, and age-adjusted mortality from heart failure (HF) **(E)**, depicted by quintiles of each variable. The arrows represent a hypothesized pathway. (Note: the states of Alaska and Hawaii are shown on the left bottom in each map for the purpose of being presented in a figure. Please refer to a U.S. map for their exact location.)

Significant variations in age-adjusted HF mortality across the 51 states were observed in men and women (Table S2 in Supplementary Material). Of the 51 states, the top three states that had the highest HF mortality rates (per 100,000 population) were Mississippi (85.0), Alabama (84.7), and Louisiana (71.2), the three states that had the lowest HF mortality were Vermont (11.7), Arizona (13.0), and Washington (15.9), respectively. Figure 2 depicts the state-level variations in the age-adjusted HF mortality rates and four risk factors of study interest. Overall, states located in the South and East had higher age-adjusted HF mortality rates [HF mortality, quantile (Q) 5:59.4–85.0 per 100,000 population], and higher age-adjusted prevalence of obesity (Q5: 30.7–34.1%), physical inactivity (Q5: 27.9–33.0%), diabetes (Q5: 10.1–11.7%), and HBP (Q5: 32.6–37.0%).

**Figure 3:**
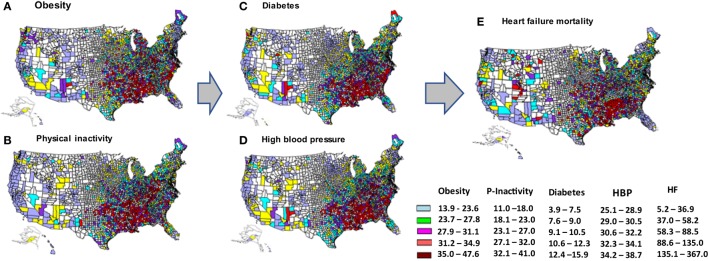
County-level variations in age-adjusted prevalence of obesity **(A)**, physical inactivity **(B)**, diabetes **(C)**, high blood pressure (HBP) **(D)**, and age-adjusted mortality from heart failure **(E)**, depicted by quintiles of each variable. The arrows represents hypothesized pathway. (Note: the states of Alaska and Hawaii are shown on the left bottom in each map for the purpose of being presented in a figure. Please refer to a U.S. map for their exact location.)

Figure 3 depicts the disproportionate distributions of HF mortality and the study risk factors of interest across the counties in the U.S. Spatial autocorrelation analysis (Table 2) indicates that the values of Moran’s *I* > 0, and Geary’s *C* > 0 and <1, which suggest that the variations in age-adjusted HF mortality, age-adjusted prevalence of obesity, physical inactivity, HBP, and diabetes by counties were positively and geographically clustered.

**Table 2:** Spatial autocorrelation analysis of the study factors across the counties.

	Moran’s *I*			Geary’s *C*		
	β	SD	*p*-Value	β	SD	*p*-Value
Obesity	0.128	0.0001	<0.0001	0.822	0.003	<0.0001
Physical inactivity	0.148	0.0001	<0.0001	0.764	0.003	<0.0001
High blood pressure	0.155	0.0001	<0.0001	0.806	0.003	<0.0001
Diabetes	0.137	0.0001	<0.0001	0.821	0.003	<0.0001
Heart failure	0.098	0.0001	<0.0001	0.894	0.003	<0.0001

*β: Spatial autocorrelation (dependency) coefficients*.

*Moran’s *I* coefficients (*β*) > 0 and Geary’s *C* coefficients > 0 and <1 indicate a positive spatial correlation (i.e., similar values cluster together in a region)*.

### Binary Analysis of the Associations Between Risk Factors and HF Mortality

Table 3 shows that the age-adjusted prevalence of diabetes, HBP, obesity and physical inactivity were positively and significantly associated with the risk of HF mortality in both sexes, and in men and women (*p* < 0.0001). Of the four risk factors, HBP shows to have the strongest correlation with the risk of HF mortality. The correlation coefficients are 0.46 (95% CI: 0.43–0.50, *p* < 0.0001) in both sexes, 0.51 (0.46–0.55, *p* < 0.0001) in men, and 0.50 (0.46–0.55, *p* < 0.0001) in women.

**Table 3:** Correlation coefficients (*r* and 95% CI) between risk factors and HF mortality.

Risk factors	HF mortality correlated with risk factors								
	In both sexes			In men			In women		
	*r*	95% CI	*p*-Value	*r*	95% CI	*p*-Value	*r*	95% CI	*p*-Value
Diabetes rate, %	0.42	0.38–0.46	<0.0001	0.46	0.41–0.51	<0.0001	0.44	0.40–0.49	<0.0001
HBP rate, %	0.46	0.43–0.50	<0.0001	0.51	0.46–0.55	<0.0001	0.50	0.46–0.55	<0.0001
Obesity rate, %	0.41	0.37–0.44	<0.0001	0.47	0.41–0.51	<0.0001	0.44	0.40–0.49	<0.0001
Phy. Inact. rate, %	0.45	0.41–0.48	<0.0001	0.50	0.45–0.54	<0.0001	0.50	0.46–0.54	<0.0001

*HF, heart failure; HBP, high blood pressure; Phy. Inact., physical inactivity*.

**Figure 4:**
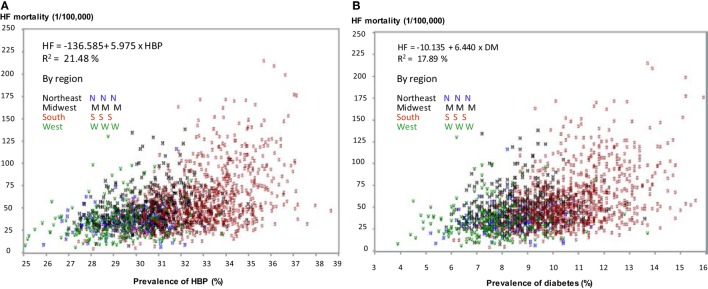
Correlation of age-adjusted prevalence of high blood pressure (HBP) **(A)** and diabetes **(B)** with age-adjusted heart failure (HF) mortality in 1,724 counties by regions.

Figure 4 depicts a linear relationship of the county-level age-adjusted prevalence of HBP (Figure 4A) and diabetes (Figure 4B) with age-adjusted HF mortality. It also illustrates that counties in the South had a higher age-adjusted prevalence of HBP and diabetes (Figures 4A,B), and higher HF mortality (in red).

**Figure 5:**
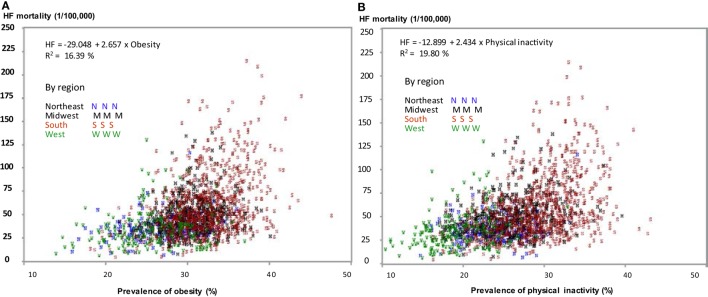
Correlation of age-adjusted prevalence of obesity **(A)** and physical inactivity **(B)** with age-adjusted heart failure (HF) mortality in 1,724 counties by regions.

Figure 5 shows a linear relationship between the county-level age-adjusted prevalence of obesity (Figure 5A), physical inactivity (Figure 5B), and age-adjusted HF mortality. It also depicts that counties in the South had a higher age-adjusted prevalence of obesity and physical inactivity (Figures 5A,B), and higher HF mortality (in red).

### Multilevel Regression Analysis

In multilevel hierarchal regression analysis (Table 4), Model 1 indicates that 42.84% of the variation [347/(347 + 463.1)*100] in HF mortality (treated as level 1 in the multilevel model) was accounted for by the differences across states (level 2) (27). Model 2 indicates that counties with an elevated prevalence of diabetes, HBP, obesity, and physical inactivity had significantly higher HF mortality, as indicated by regression coefficients, β = 3.05 (95% CI: 2.21–3.90, *p* < 0.001), 3.80 (2.30–5.29, *p* < 0.0001), 1.20 (0.89–1.51, *p* < 0.0001), and 1.47 (1.17–1.76, *p* < 0.0001) for diabetes, HBP, obesity, and physical inactivity, respectively. Model 3 shows that the effects of the four risk factors on HF mortality were attenuated after adjustment for demographic and socioeconomic variables (male-to-female ratio, % of African Americans, and poverty rate). With a further adjustment for obesity and physical inactivity, Model 4 shows that the effects of diabetes on HF mortality become non-statistically significant (*p* = 0.121). The effect of HBP on HF mortality decreased as well (β = 3.56 in Model 3 and 2.41 in Model 4, a 32.3% reduction), but it remains statistically significant (*p* = 0.003). It suggests that for an estimated every 1% increase in the prevalence of HBP, HF mortality increases by 2.41 units. The values of AIC (with a smaller value) and Pseudo *R*^2^ (with a larger value) suggest that the models with adjusting covariates have a better fitness of the regression between the risk factors and HF mortality than the other models (30, 31). For example, for Models 3 and 4, in the relationship between diabetes and HF mortality, the values of Pseudo *R*^2^ from 3.64 to 4.68% represents the proportional reduction in residual variation from Model 3 to Model 4.

**Table 4:** Multilevel hierarchical linear regression models of the effects of risk factors on risk of heart failure mortality.

	Model 1			Model 2			Model 3			Model 4		
	β	SE	*p*	β	SE	*p*	β	SE	*p*	β	SE	*p*
**Diabetes**												
Fixed effects												
Intercept	45.0	2.8	<0.0001	17.87	4.6	0.0001	−105.31	71.0	0.068	−131.3	75.4	0.088
Diabetes				**3.05**	0.4	**<0.0001**	**2.70**	0.5	**<0.0001**	1.00	0.6	0.121
Random effects												
Residual (Cty)	463.1	16.0	<0.0001	454.1	15.7	<0.0001	446.3	14.4	<0.0001	441.5	15.3	<0.0001
Intercept (state)	347.0	75.8	<0.0001	255.9	57.6	<0.0001	243.4	45.6	<0.0001	417.4	51.2	<0.0001
Model fit, AIC	15,609			15,562			15,433			15,411		
Pseudo *R*^2^, %				1.95			3.64			4.68		
V. acc for by state, %	42.8			36.0			35.3			48.6		

**High blood pressure (HBP)**												
Fixed effects												
Intercept				69.97	23.4	0.005	−261.59	79.4	0.002	−249.6	78.8	0.003
HBP				**3.80**	0.8	**<0.0001**	**3.56**	0.7	**<0.0001**	**2.41**	0.8	**0.003**
Random effects												
Residual (Cty)				464.1	16.1	<0.0001	451.6	15.7	<0.0001	442.2	15.4	<0.0001
Intercept (state)				220.8	51.5	<0.0001	185.9	44.8	<0.0001	183.5	44.3	<0.0001
Model fit, AIC				15,554			15,404			15,369		
Pseudo *R*^2^, %				−0.2			2.5			4.5		
V. acc for by state, %				32.2			29.2			29.3		

**Obesity**												
Fixed effects												
Intercept				10.19	5.2	0.052	−99.4	81.0	0.23			
Obesity				**1.20**	0.2	**<0.0001**	**0.96**	0.2	**<0.0001**	Adjusted		
Random effects												
Residual (Cty)				451.6	15.6	<0.0001	445.2	15.5	<0.0001			
Intercept (state)				267.0	59.7	<0.0001	257.6	59.4	<0.0001			
Model fit, AIC				15,556			15,433					
Pseudo *R*^2^, %				2.5			3.9					
V. acc for by state, %				37.2			36.6					

**Physical inactivity**												
Fixed effects												
Intercept				8.85	4.5	0.048	−122.8	76.2	0.113			
Physical inactivity				**1.47**	0.2	**<0.0001**	**1.19**	0.2	**<0.0001**	Adjusted		
Random effects												
Residual (Cty)				444.5	15.4	<0.0001	442.4	15.4	<0.0001			
Intercept (state)				235.7	53.2	<0.0001	223.1	52.5	<0.0001			
Model fit, AIC				15,524			15,417					
Pseudo *R*^2^, %				4.02			4.5					
V. acc for by state, %				34.6			33.5					

*Model 1: test for variation accounted for by states*.

*Model 2: test fixed effect of risk factor on HF mortality*.

*Model 3: adjusted for male/female ratio, % of African American, and poverty rate in adults aged 18 and older*.

*Model 4: adjusted for male/female ratio, % of African American, poverty rate in adults, % of obesity, and % of physical inactivity*.

*AIC, Akaike information criterion; BIC, Bayesian information criterion*.

*Pseudo R^2^ represents the proportion reduction in residual variance between two nested models*.

For example, analysis of the association between diabetes and HF, M1,2 Pseudo *R*^2^ = (463.1 − 454.1)/463.1*100 = 1.95%. M1,3 Pseudo *R*^2^ = (463.1− 446.25)/463.1*100 = 3.64%.

*V. acc for by state: variation in HF mortality accounted for by states*.

*Significant values for the associations between risk factors and HF are in bold*.

## Discussion

Three main findings of this study are (1) it is one of the first studies that maps the burden of HF mortality and examines the associations between four preventable risk factors and the risk of HF mortality across the counties and states of the U.S. It highlights that an estimate of 42.8% of the variations in HF mortality is accounted for by the differences across 50 states. (2) The study further highlights that the geographic disparity in HF mortality is significantly associated with the disproportionate distributions of the prevalent obesity, physical inactivity, diabetes and HBP across the counties and states. These associations are independent of gender, race/ethnicity and socioeconomic status (assessed by poverty rate). (3) After controlling for obesity and physical inactivity, the effects of diabetes and HBP on HF mortality are hugely attenuated, which further confirms the importance of changes in behavior risk factors in disease control.

An increasing burden of HF has been reported by several studies, including our own reports (3, 5, 33–36). Few studies paid attention to the geographic disparities in HF. Most previous studies focused on individual risk factors at the personal level, which are undoubtedly important to provide information to precision medicine at an individual level (37–40). However, it is known that health policy and health programs are made and implemented at county- and state levels in the U.S. Therefore, in addition to having the studies at a personal level, studies at the county and state levels are needed to provide important evidence for making better policy. Findings from this study further address the geographic disparity in HF mortality and provide new insights into the disease and risk factors control, which is critical in evaluating, improving and moving toward the goal of the healthy counties and states in the nation.

In the multilevel hierarchal regression analysis, findings from Model 3 highlight the significant independent effects of each risk factor on the risk of HF mortality. In Model 4, the association between diabetes and HF mortality became non-significant after further adjusting for obesity and physical inactivity. Given a high correlation of obesity and physical inactivity with diabetes (data not shown), it is very likely that this non-significant result is largely explained by the adjustment for a possible pathway of these two factors for the risk of diabetes, then subsequently to the risk of HF. It should be noted that although this study does not necessary to interpret any cause inference relationship due to the nature of the ecological study design, the pathway of obesity and physical inactivity with the risk of diabetes and HBP, and diabetes and HBP as independent risk factors for cardiovascular diseases are well established (2). Given a relatively cost-effect approach of controlling obesity and improving physical activity for multiple diseases prevention, findings from the study highlight the importance of behavior changes and primary health care for controlling HF.

The mechanisms by which the behavioral-related and disease risk factors cause the development of HF and risk of HF mortality have been established at personal levels. For example, the associations of obesity and physical inactivity with an increased risk of HF may go through their effects on an increased risk for HBP and diabetes. However, at a population level, the geographic variations in HF mortality may indicate an even wider range of risk factors, including tobacco control and health-care support systems. Presently, we did not examine the associations between these factors and risk of HF mortality because of the lack of the relevant and valid data. Further studies are needed to confirm the current findings and extend the current work.

There are several advantages of this study. First, to the best of our knowledge, the study using a spatial and ecological study design in HF mortality study at the county-level, has the largest sample size (1,723 counties from 51 states), and the key measures of the study variables of interest (exposures and outcomes) are defined and examined using standard approaches by the U.S. CDC. Second, the study addresses two important components of risk factors for HF mortality, one related to the health behaviors, and the other related to the common diseases controlled by the primary health-care settings. Third, we applied multilevel regression analysis technique, which addresses the variations accounted for by states and we can further precisely test the associations between county-level exposures and HF mortality withholding the hierarchical effect due to the variation accounted for by the states.

It should be noticed that there are several limitations when interpreting the results of the study. First, potential ecologic bias may have occurred when we analyzed the data across the county- and state levels. The risk of HF mortality and the prevalent risk factors of interest at county- and state levels is not necessary to represent an individual risk of HF. In the study, we applied an ecologic analysis approach with aims to addressing geographic health disparity in HF mortality and its possible reduction or elimination by examining its associations with four most common and preventable risk factors across the county- and state levels. Meanwhile, we were unable to address race/ethnicity differences across the counties in the study, because most counties had a small proportion of minority populations, except for people living in the southern regions. Certainly, further studies are needed to focus on race/ethnicity disparities in HF. Second, several other risk factors for HF, such as cigarette smoking, dietary patterns, and health-care services indicators are not included because of the lack of age-specific and county-level data in detail. Therefore, findings from the study are subjected to represent the involved study factors of interest only. Like any other studies, the magnitudes of the associations between risk factors and outcomes of interest largely depend on what variables are included in multivariable and multilevel models. Therefore, findings of the study are not necessary to be generalized for a study that contains different study variables. However, a basic trend of these associations is expected in most studies. Last, but not least, age- and sex-adjusted rates were not available in the study because age-specific outcomes (HF) and risk factors (obesity, physical inactivity, and diabetes) were reported in groups by sex and county (i.e., there are no individual observations) in the CDC WONDER and CDC County Data system. To keep the limitation in mind, we test and present our results by sex whenever it is possible. In our multilevel regression models, to examine and present the sex-combined analysis, we adjusted county-level male-to-female ratio and percent of African Americas in Models 3 and 4.

Although several limitations may exist, the present analysis is one of the first large-scale studies across the counties that address the geographic health disparities in HF mortality and its associations with risk factors across the nation. Findings from the study emphasize that the four most common and preventable risk factors both related to health behaviors and the health-care system at county-level significantly predict age-adjusted mortality from HF. These geographic differences need to be paid specific attention in HF prevention and control.

## Author Contributions

LL: did the study design, conducted data analysis, and drafted the manuscript. XY: participated in the study design, assisted in doing visualization and spatial analysis using GIS and SAS, and gave comments on the draft manuscript. MC, HJ, HE and AH: participated in the study design and gave comments on the draft manuscript.

## Conflict of Interest Statement

The authors declare that the research was conducted in the absence of any commercial or financial relationships that could be construed as a potential conflict of interest.
